# Characterization of a new fiber-reinforced flowable composite

**DOI:** 10.1007/s10266-018-0405-y

**Published:** 2019-01-08

**Authors:** Lippo Lassila, Eija Säilynoja, Roosa Prinssi, Pekka Vallittu, Sufyan Garoushi

**Affiliations:** 1 Department of Biomaterials Science and Turku Clinical Biomaterial Center-TCBC, Institute of Dentistry, University of Turku, Turku, Finland; 2 Research Development and Production Department, Stick Tech Ltd-Member of GC Group, Turku, Finland; 3 City of Turku Welfare Division, Oral Health Care, Turku, Finland

**Keywords:** Short fiber-reinforced flowable resin composite, Fracture toughness, Depth of cure, Bulk fill resin composite, Physical properties

## Abstract

This study aimed to evaluate certain physical properties including surface wear of a new experimental short fiber-reinforced flowable resin composite (SFRC) in comparison with different commercial flowable bulk fill resin composites (SDR, Tetric EvoFlow Bulk Fill, Filtek Bulk Fill Flowable and Estelite Bulk Fill Flow). The following properties were examined according to ISO standard: flexural strength, flexural modulus, fracture toughness, water sorption, volumetric shrinkage, and depth of cure. Degree of conversion (DC%) was determined by FTIR spectrometry. A wear test was conducted with 15000 chewing cycles using a dual-axis chewing simulator. Wear depth was measured by a three-dimensional (3D) noncontact optical profilometer. Scanning electron microscopy was used to evaluate the microstructure of SFRC. Data were statistically analyzed with analysis of variance ANOVA (*p* = 0.05). SFRC exhibited the highest fracture toughness (2.8 MPa m^1/2^) and flexural strength (146.5 MPa) values (*p* < 0.05) and the greatest depth of cure (5 mm) and lowest wear depth (18.2 µm) among the flowable bulk fill materials tested. SDR showed the lowest volumetric shrinkage percentage (2.9%), while the other resin composites had comparable volumetric shrinkage values (*p* > 0.05). The new short fiber-reinforced flowable resin composite differed significantly in its measured fracture toughness compared to the tested flowable bulk fill resin composites.

## Introduction

The use of light-cured resin composites for restoring cavities in stress-bearing posterior teeth has increased rapidly in recent years [1]. Especially, the improved handling characteristics have resulted in an increased popularity of flowable conventional and flowable bulk-fill resin composites. Beside the ability to bond to hard tooth tissues, mediated by adhesive systems, they feature the advantage of good esthetics and are less expensive compared with cast gold and ceramic inlays. However, insufficient material properties limited the success of composite restorations in high stress bearing areas [2, 3]. Fracture within the body and at the margins of restorations and polymerization shrinkage have been cited as major problems regarding the failure of posterior composites [4]. The fracture-related material properties, such as fracture resistance, deformation under occlusal load, and the marginal degradation of materials have usually been evaluated by the determination of the basic material parameters of flexural strength and fracture toughness [5]. Fracture toughness values are dependent on the mechanical properties and chemical composition of the individual component contained in the restorative material. A material which has high fracture toughness has the ability to better resist crack initiation and propagation. Consequently, the property of fracture toughness and flexural strength becomes important criterions in a dental materials’ longevity [6, 7].

Depending on the studies, volumetric shrinkage of the resin composites averages from 1.5 to 6% [8]. Such shrinkage induces contraction stress at the interface between resin composite and walls of cavity leading to gap formation and a predisposition for secondary caries. Different measurement techniques were used to follow and to understand this phenomenon, including the mercury dilatometric technique, the bonded-disc technique, strain-gage methods, cuspal deflection method, and shrinkage stress tests [9–11].

Several manufacturers have developed posterior “bulk fill” flowable resin composites which claimed that they can be applied to the cavity at a thickness of 4 mm with enhanced curing, shrinkage, mechanical, and wear properties. A problem associated with using light cured resin composite directly in the posterior region is the decrease in curing-light intensity with depth in the material. The intensity of light at a given depth and for a given irradiance period is a critical factor in determining the extent of reaction of monomer into polymer, typically referred to as the degree of conversion, and significantly associated with values of mechanical properties, biocompatibility, color stability and would, therefore, be expected to be associated with clinical success of the restoration [12, 13].

Studies have been carried out with this flowable bulk fill class of materials; however, the results reported in the literature vary considerably. Some authors show similar or higher mechanical performance and lower polymerization shrinkage compared to conventional resin composites [14–16]. Others describe a significant decrease of monomer conversion and mechanical performance in bulk-fill resin composites at 4-mm thickness or higher volumetric shrinkage than that of conventional resin composites [17–19].

Recently, a new experimental short fiber-reinforced flowable resin composite (SFRC) was introduced as a restorative material. This resin composite is intended to be used as dentine replacing material in high stress bearing areas especially in large cavities of vital and non-vital posterior teeth. It consists of a combination of a resin matrix, randomly orientated glass microfibers and inorganic silanated particulate fillers. Thus, the aim of this study was to investigate the physical properties (i.e., flexural strength, flexural modulus, fracture toughness, water sorption, degree of conversion, depth of cure, and polymerization shrinkage) and wear of SFRC compared to certain commonly used flowable bulk fill resin composites.

## Materials and methods

The commercial and experimental flowable resin composites, associated lot numbers, and components are shown in Table 1.

**Table 1:** Flowable bulk fill resin composites investigated and their composition

Material (code)	Manufacturer (lot no.)	Matrix composition		Inorganic filler content
SureFil (SDR)	Dentsply, Milford, USA (161116)		TEGDMA, EBPADMA	Barium borosilicate glass 68 wt%, 44 vol%
Filtek Bulk Fill Flowable (Filtek)	3M/ESPE, St. Paul, MN, USA (N827902)		Bis-GMA, EBPADMA, UDMA	Ceramic and ytterbium trifluoride 64 wt%, 42 vol%
Tetric EvoFlow Bulk Fill (Tetric)	Ivoclar Vivadent Schaan, Liechtenstein (P63316)		Bis-GMA, EBPADMA	Barium glass, ytterbium trifluoride 68.2 wt%, 46.4 vol%
Estelite Bulk Fill Flow (Estelite)	Tokuyama Dental Corp, Ibaraki, Japan (004E06)		Bis-MPEPP, TEGDMA, Bis-GMA	Silica-zirconia 70 wt%, 56 vol%
Short fiber flowable composite (SFRC)	GC Corp, Tokyo, Japan (experimental)		Bis-EMA, TEGDMA, UDMA	Short glass fiber (200–300 µm and Ø7 µm) + barium glass 70 wt%

*Bis-GMA* bisphenol-A-glycidyl dimethacrylate, *TEGDMA* triethylene glycol dimethacrylate, *UDMA* urethane dimethacrylate, *EBPADMA* ethoxylated bisphenol A dimethacrylate, *Bis-EMA* ethoxylated bisphenol-A-dimethacrylate, *Bis-MPEPP* bisphenol A polyethoxy methacrylate, *wt*% weight percentage, *vol*% volume percentage

### Flexural strength and modulus of elasticity

Three-point bending test specimens (2 × 2 × 25 mm^3^) were made from each tested resin composite. Bar-shaped specimens were made in a half-split stainless steel mold between transparent Mylar sheets. Polymerization of the resin composite was done using a hand light-curing unit (Elipar S10, 3M ESPE, St. Paul, MN, USA) for 20 s in five separate overlapping portions from both sides of the metal mold. The wavelength of the light was between 430 and 480 nm and light intensity was 1600 mW/cm^2^ (Marc Resin Calibrator, BlueLight Analytics Inc., Canada). The specimens from each group (*n* = 8) were stored dry at 37 °C for 48 h before testing. A three-point bending test was conducted according to the ISO 4049 (test span: 20 mm, cross-head speed: 1 mm/min, indenter: 2 mm diameter). All specimens were loaded in a material testing machine (model LRX, Lloyd Instrument Ltd, Fareham, England) and the load-deflection curves were recorded with PC-computer software (Nexygen 4.0, Lloyd Instruments Ltd, Fareham, England).

Flexural strength (*ơ*_*f*_) and flexural modulus (*E*_*f*_) were calculated from the following formula [20]:


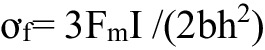

$${E_f}=S{I^3}/(4b{h^3}),$$

where *F*_m_ is the applied load (*N*) at the highest point of the load-deflection curve, *I* is the span length (20 mm), *b* is the width of the test specimens and *h* is the thickness of the test specimens. *S* is the stiffness (N/m) *S* = *F*/*d*, and *d* is the deflection corresponding to load *F* at a point in the straight-line portion of the trace.

### Fracture toughness

Single-edge-notched-beam specimens (2.5 × 5 × 25 mm^3^) according to adapted ISO 20795-2 standard method (ASTM 2005) were prepared to determine the fracture toughness [21]. A custom-made stainless steel split mold was used, which enabled specimen removal without force. Accurately designed slot was fabricated centrally in the mold extending until its mid-height, which enabled central location of the notch and optimization of the crack length (*x*) to be half of specimen’s height. The resin composite was inserted into the mold placed over a Mylar-strip-covered glass slide in one increment. Before polymerization, a sharp and centrally located crack was produced by inserting a straight edged steel blade into the prefabricated slot. Polymerization of the resin composite was carried out for 20 s in five separate overlapping portions. The upper side of the mold was covered with a Mylar strip and glass slide from both sides of the blade, before being exposed to the polymerization light. Upon the removal from the mold, each specimen was polymerized also on the opposite side. The specimens from each group (*n* = 6) were stored dry at 37 °C for 48 h before testing. The specimens were tested in three-point bending mode, in a universal material testing machine at a crosshead speed of 1.0 mm/min.

The fracture toughness was calculated using the equation: ${K_{\hbox{max} }}=f(x)[PL/(B{W^{3/2}})]\sqrt {10 - 3}$, where: $f(x)=3/2{x^{1/2}}[1.99 - x(1 - x)(2.15 - 3.93x+2.7{x^2})]/2(1+2x){(1 - x)^{3/2}}$ and 0 < *x* < 1 with *x* = *a*/*W*. Here, *P* is the maximum load in Newton (N), *L* is the span length (20 mm), *B* is the specimen thickness (mm), *W* is the specimen width (depth) in mm, *x* is a geometrical function dependent on *a*/*W*, and *a* is the crack length in mm.

### Depth of cure

The depth of cure analysis for the tested materials was performed according to ISO standard 4049 with 10 mm high cylinder [22]. The mold was placed on a glass slide covered by a Mylar strip. The mold was then filled in bulk with one of the tested resin composites. The top side of the mold was covered with a second Mylar strip and the resin material made flush with the mold by use of a second glass slide. The specimens (*n* = 3) were polymerized from the top of the cylinder mold with a hand-light curing unit according to manufacturer recommendation using a light source (Elipar S10, 3M ESPE, St. Paul, MN, USA) with an irradiance of 1600 mW/cm^2^ (Marc Resin Calibrator, BlueLight Analytics Inc., Canada). As soon as the curing was over, the material was pressed out from the mold and using a plastic spatula, the part which had not been polymerized was removed. Then, the remaining cured part was measured with a digital caliber with accuracy of ± 0.1 mm and the given value was divided by two. This value was recorded as the depth of cure for each specimen.

### Degree of conversion

Degree of conversion (DC%) during and after the photoinitiation of polymerization was monitored by Fourier transform infrared spectroscopy (FTIR) (Spectrum One, Perkin-Elmer, Beaconsfield Bucks, UK) with an attenuated total reflectance (ATR) accessory. Resin composites were analyzed in a mold that was 1.5 mm in thick and 4.5 mm in diameter. First, the spectrum of the unpolymerized sample was placed in the mold and measured. Then, the sample was irradiated through an upper glass slide for 40 s with a visible light-curing unit (Elipar S10, 3M ESPE, St. Paul, MN, USA) producing an average irradiance of 1600 mW/cm^2^ (Marc Resin Calibrator, BlueLight Analytics Inc., Canada). The sample was scanned for its FTIR spectrum after being irradiated. The DC% was calculated from the aliphatic C=C peak at 1638 cm^−1^ and normalized against the aromatic C=C peak at 1608 cm^−1^ according to the following formula:

$${\text{DC}}\% =\left[ {1 - \frac{{{C_{{\text{aliphatic}}}}/{C_{{\text{aromatic}}}}}}{{{U_{{\text{aliphatic}}}}/{U_{{\text{aromatic}}}}}}} \right] \times 100\% ,$$

where is the *C*_aliphatic_ is the absorption peak at 1638 cm^−1^ of the cured specimen, *C*_aromtic_ is the absorption peak at 1608 cm^−1^ of the cured specimen, *U*_aliphatic_ is the absorption peak at 1638 cm^−1^ of the uncured specimen, and *U*_aromatic_ is the absorption peak at 1608 cm^−1^ of the uncured specimen.

The fraction of remaining double bonds for each spectrum was determined by standard baseline techniques using the comparison of maximum heights of aliphatic and reference peaks for calculations. For each resin composite, five trials were performed.

### Volumetric shrinkage

The specimens’ densities (*n* = 5) were measured to determine volume shrinkage according to Archimedes’ principle (ISO 17304) with a commercial density determination kit of the analytical balance (XS105, Mettler Toledo, Greifensee, Switzerland). The mass of the specimen was weighed in air and water, and density was calculated according to the equation:

$$D=\frac{{{M_1} \times {D_{\text{w}}}}}{{{M_1} - {M_2}}},$$

where *D* is the density of the sample, *M*_1_ is the mass of the sample in air, *M*_2_ is the mass of the sample in water, and *D*_w_ is the density of water at the measured temperature. For each resin composite, six trials were performed, respectively, to calculate the densities of polymerized and unpolymerized samples. The volume shrinkage (VS) was expressed in % and calculated from the densities according to the equation:

$${\text{VS}}=\frac{{{D_{\text{c}}} - {D_{\text{u}}}}}{{{D_{\text{c}}}}} \times 100\% ,$$

where *D*_u_ is the density of the unpolymerized sample and *D*_c_ is the density of the polymerized sample.

### Water sorption

Water sorption for each material was measured from seven specimens which were stored in 120 ml of water for 36 days at 37 °C. The dry weight (*m*_d_) of the specimens was measured with a balance (Mettler A30, Mettler Instrument Co., Highstone, Nj, USA), with an accuracy of 0.1 mg. During water immersion, specimen weight (*m*_*w*_) was measured at 1, 2, 3, 4, 10, 14, 28, 31, and 36 days. Water uptake was calculated as follows:

$${\text{Water uptake}}\% =({m_w} - {m_{\text{d}}})/{m_{\text{d}}} \times 100\% .$$

### Two-body wear

Two specimens of each resin composite were prepared in an acrylic resin block for localized wear testing. Longitudinal cavities (20 mm length × 10 mm width × 3 mm depth) were prepared in and then resin composites were placed in one increment into the prepared cavities and covered with Mylar strips and glass slides before light irradiated for 20 s in five separate overlapping portions. The surfaces were then polished flat using a sequence of #1200- to #4000-grit silicon carbide papers. After 1 day of water storage (37 °C), 2-body wear test was conducted using the chewing simulator CS-4.2 (SD Mechatronik, Feldkirchen–Westerham, Germany) which has two chambers simulating the vertical and horizontal movements simultaneously with water. Each of the chambers consisted of an upper sample holder that can fasten the loading tip (antagonistic) with a screw and a lower plastic sample holder in which the resin composite specimen was embedded. The manufacturer’s standard loading tips (Steatite ball, Ø 6 mm) were embedded in acrylic resins in the upper sample holders, and were then fixed with a fastening screw. A weight of 2 kg, which is comparable to 20 N of chewing force, and 15,000 loading cycles with frequency of 1.5 Hz were used.

The wear patterns (*n* = 6) on the surface of each specimen were profiled with 3D optical microscope (Bruker Nano GmbH, Berlin, Germany) using Vision64 software. The maximum wear depth values (µm), representing the average of the lowest or deepest points of all profile scans, were calculated from different points.

### Microscopic analysis

Scanning electron microscopy (SEM, JSM 5500, Jeol Ltd., Tokyo, Japan) provided the characterization of the microstructure, fractographic, and wear surface examination of the SFRC resin composite. Specimens (*n* = 3) from fracture toughness test (single-edge-notched-beam specimens) and two-body wear test (specimen with wear patterns on the surface) were stored in desiccator for 1 day. Then, they were coated with a gold layer using a sputter coater in vacuum evaporator (BAL-TEC SCD 050 Sputter Coater, Balzers, Liechtenstein) before the SEM examination. SEM observations were carried out at an operating voltage of 8–15 kV.

### Statistical analysis

The data were statistically analyzed with SPSS version 23 (SPSS, IBM Corp.) using analysis of variance (ANOVA) at the *p* < 0.05 significance level followed by a Tukey HSD post hoc test to determine the differences between the groups.

## Results

**Fig. 1:**
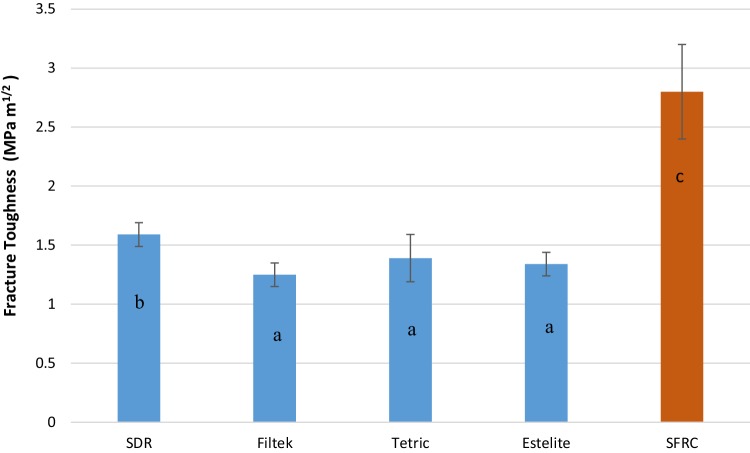
Bar graph illustrating mean fracture toughness (KIC) and standard deviation (SD) of investigated materials. The same letters inside the bars represent non-statistically significant differences (*p* > 0.05) among the groups

**Fig. 2:**
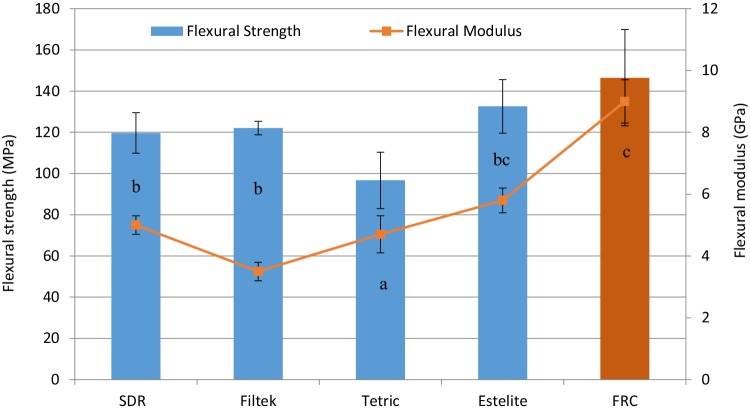
Bar graph illustrating means flexural strength (MPa), flexural modulus (GPa), and standard deviation (SD) of investigated materials. The same letters inside the bars represent non-statistically significant differences (*p* > 0.05) among the groups

**Fig. 3:**
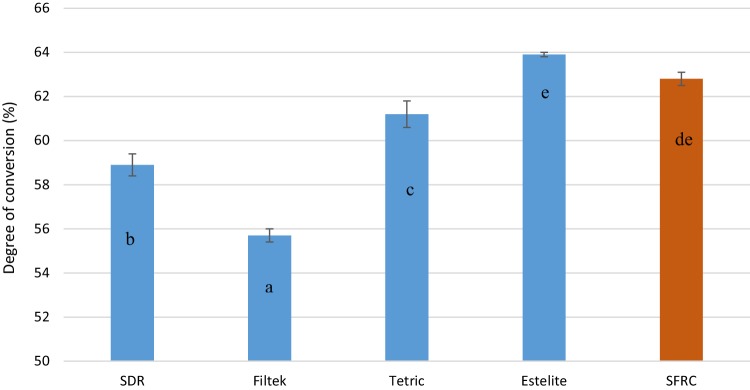
Degree of conversion percentage (DC%) measured at the bottom surface of investigated materials. The same letters inside the bars represent non-statistically significant differences (*p* > 0.05) among the groups

**Fig. 4:**
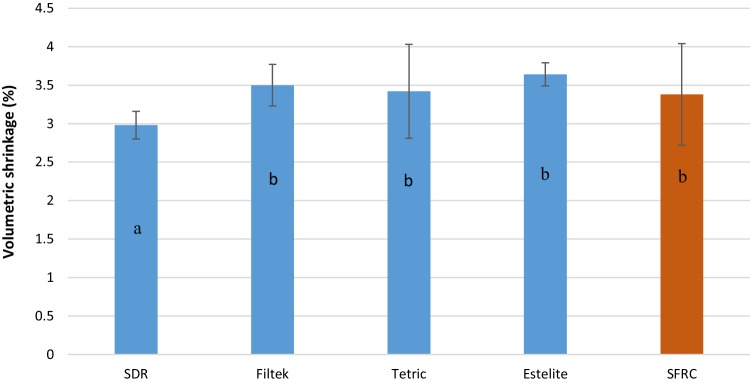
Bar graph illustrating means volumetric shrinkage (%) and standard deviation (SD) of investigated materials. The same letters inside the bars represent non-statistically significant differences (*p* > 0.05) among the groups

**Fig. 5:**
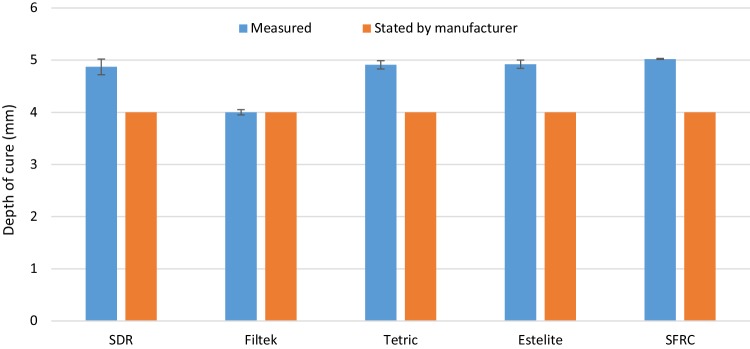
Bar graph illustrating the measured curing depth (after recommended curing time) and the stated by manufacturer (mm) of investigated materials

The mean values of fracture toughness, flexural strength, flexural modulus, DC%, and volumetric shrinkage for tested resin composites with standard deviations (SD) are summarized in Table 2 and Figs. 1, 2, 3 and 4. ANOVA revealed that the SFRC flow resin composite had a statistically significantly higher fracture toughness (2.8 MPa m^1/2^), flexural strength (146.5 MPa), and flexural modulus (9 GPa) than all other tested resin composites (*p* < 0.05). Filtek Bulk Fill Flowable presented the lowest fracture toughness (1.2 MPa m^1/2^), flexural modulus (3.5 GPa), and DC% (55.7) values among the materials tested (*p* < 0.05). Estelite Bulk Fill Flow had the highest DC% (63.9), which was not significantly different from SFRC (62.8) (*p* > 0.05). SDR showed the lowest volumetric shrinkage percentage (2.9%), while the other resin composites had comparable volumetric shrinkage values (*p* > 0.05). Curing depth of SFRC was found to be 5 mm which is similar to other tested flowable bulk fill resin composites except Filtek Bulk Fill which had the lowest depth of cure (4 mm) (Fig. 5).

**Table 2:** Mean values (± SD) of fracture toughness (FT), flexural strength (FS), flexural modulus (FM), degree of conversion (DC), volumetric shrinkage (VS), depth of cure (DOP), water sorption (WS), and wear depth (WD)

Material	FT (MPa m1/2)	FS (MPa)	FM (GPa)	DC (%)	VS (%)	DOC (mm)	WS (%)	WD (mµ)
SDR	1.6 ± 0.1^b^	120 ± 9.8^b^	5 ± 0.3^b^	58.9 ± 0.5^b^	2.9 ± 0.1^a^	4.8 ± 0.1	0.6 ± 0.04	31.3 ± 2.3^bc^
Filtek	1.2 ± 0.1^a^	122 ± 3.3^b^	3.5 ± 0.3^a^	55.7 ± 0.3^a^	3.5 ± 0.2^b^	4 ± 0.05	0.9 ± 0.06	34.9 ± 4.6^c^
Tetric	1.4 ± 0.2^a^	97 ± 13^a^	4.7 ± 1.6^b^	61.2 ± 0.6^c^	3.4 ± 0.6^b^	4.9 ± 0.08	0.4 ± 0.03	19 ± 2^a^
Estelite	1.3 ± 0.1^a^	133 ± 13^bc^	5.8 ± 0.4^c^	63.9 ± 0.1^e^	3.6 ± 0.1^b^	4.9 ± 0.08	1.1 ± 0.2	35 ± 2.7^c^
SFRC	2.8 ± 0.4^c^	146.5 ± 23^c^	9 ± 0.7^d^	62.8 ± 0.3^de^	3.3 ± 0.6^b^	5 ± 0.01	0.5 ± 0.1	18.2 ± 4.1^a^

Same superscript letter above the values indicates groups that were statistically similar (*p* > 0.05)

**Fig. 6:**
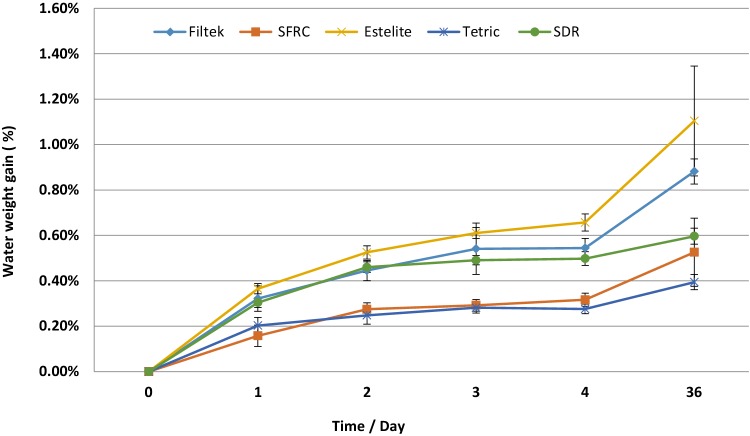
Water sorption (%wt gain) of investigated materials during 36 days of storage in water at 37 °C

The amount of water sorption (Fig. 6) after 36 days was the highest in Estelite Bulk Fill Flow (1.1 wt%) and the lowest in Tetric EvoFlow Bulk Fill (0.4 wt%) followed by SFRC (0.5 wt%).

**Fig. 7:**
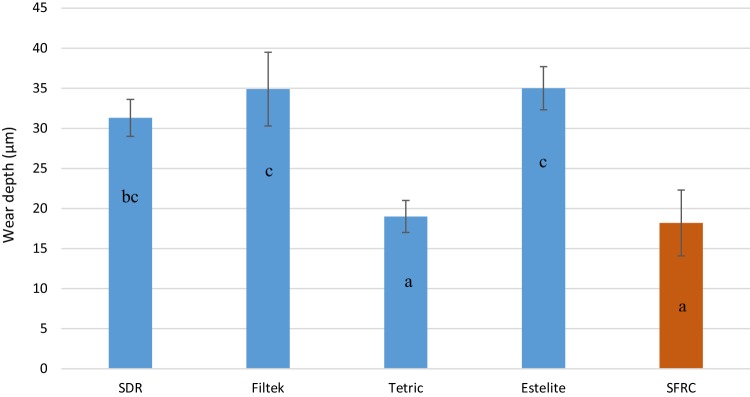
Bar graph illustrating mean wear depth (micron) and standard deviation (SD) of investigated materials after 15,000 cycles of 2-body wear test. The same letters inside the bars represent non-statistically significant differences (*p* > 0.05) among the groups

Figure 7 displays the mean values for wear depth recorded for each resin composite after 15,000 chewing simulation cycles. Lowest wear values were found for SFRC and Tetric EvoFlow Bulk Fill resin composites (18.2 and 19 µm) (*p* < 0.05).

**Fig. 8:**
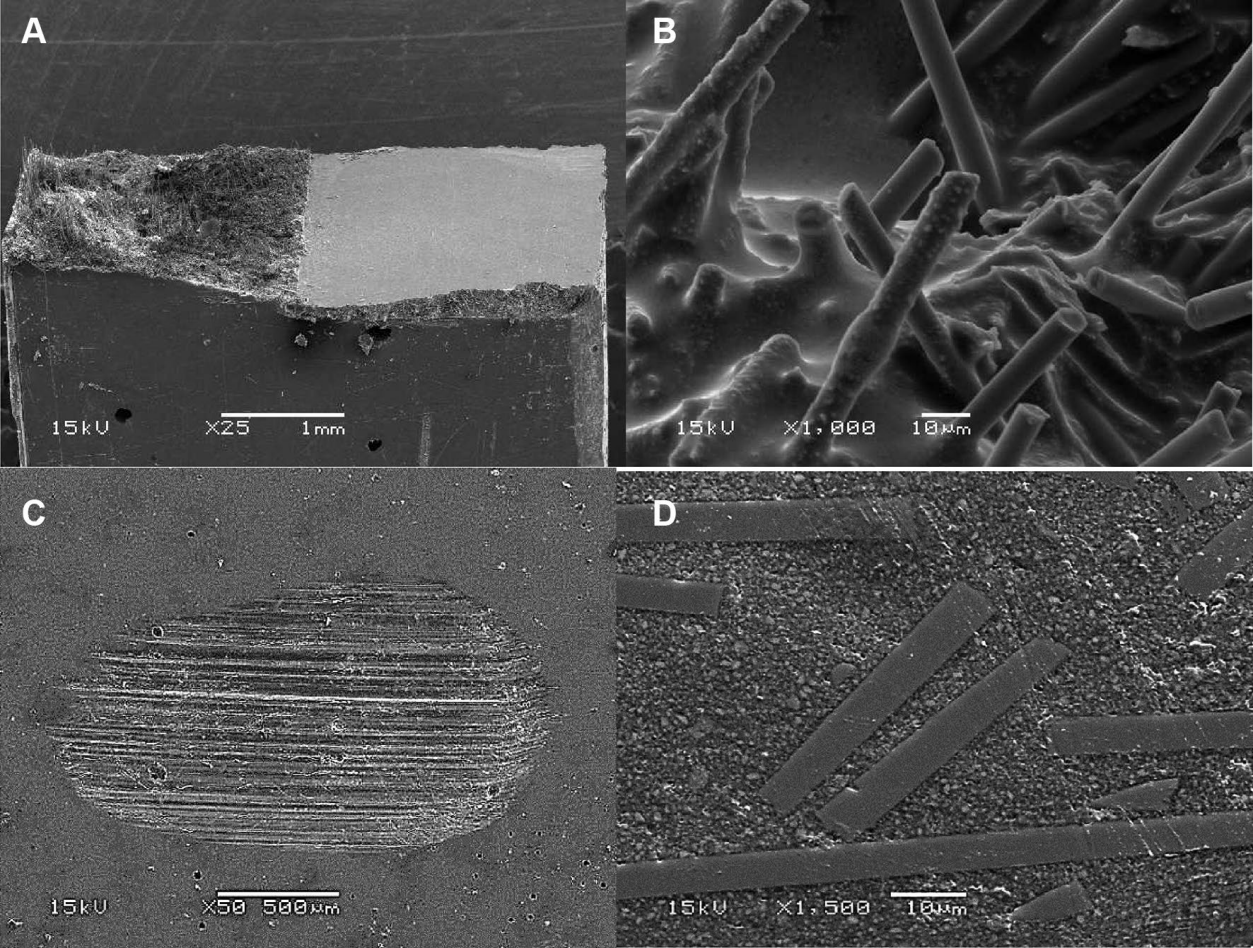
SEM photomicrographs of the SFRC material showing pull-out of fibers from fractured single-edge-notched-beam specimen (**a**). Random orientation of microfibers in the resin matrix (**b**) and wear facet after 15,000 cycles of 2-body wear test (**c, d**)

SEM analysis of the tested single-edge-notched-beam specimens showed random orientation and protruded (pullout) fiber ends at fracture surfaces of SFRC composite matrices (Fig. 8a). In addition, it presented the good wettability of microfibers within the composite matrix (Fig. 8b).

Representative SEM images of the wear facets for SFRC resin composite are shown in Fig. 8c, d. Specimens showed that microfibers were fractured into small pieces and polished down together with the composite matrix.

## Discussion

In the present study, the new experimental short fiber-reinforced flowable resin composite (SFRC) exhibited significantly higher fracture toughness (2.8 MPa m^1/2^) and flexural strength (146.5 MPa) values than all other tested materials. This is in accordance with Lassila et al. and Shouha et al. studies, which showed superior fracture toughness and flexural properties of experimental short fiber reinforced flowable resin composite compared to conventional particulate filler resin composites [7, 23]. The toughening mechanisms provided by the microfibers are the result of their ability to deflect crack propagation, to bridge, to resist the opening and propagation of the crack, consequently inducing a closure force on the crack [24]. The reinforcing effect of the fiber fillers is based on stress transfer from polymer matrix to fibers but also the behavior of individual fiber as a crack stopper. A previous study by Garoushi et al. showed how short fiber fillers could stop the crack propagation and provide an increase in fracture resistance of the resin composite [25].

The fracture toughness of a material is a measure of how well that material hinders the progress of a crack or flaw under load. Fiber impedes the extension of a crack and develops interlocking bridges behind the progressing crack dissipating energy by fiber pullout resulting in graceful rather than catastrophic failure (Fig. 8a). This might be due to the random orientation of microfibers in resin matrix and the formation of a fiber network (Fig. 8b), which seemed to have enhanced the ability of the material to resist the fracture propagation, as well as to reduce the stress intensity at the crack tip from which a crack propagates in an unstable manner. As a consequence, an increase in flexural properties and fracture toughness can be expected. Interestingly, the addition of microfibers into the resin matrix and forming fiber network did not effect the flowability of the material. To authors’ knowledge, this type of fiber reinforcing system is not well documented in the literature.

Aspect ratio, critical fiber length, fiber loading, and fiber orientation are the main factors that could improve or impair the mechanical properties of SFRC [26]. Aspect ratio is the fiber length to fiber diameter ratio (*l*/*d*). It affects the tensile strength, flexural modulus, and the reinforcing efficiency of the SFRC [27]. The microfibers used in this study had an aspect ratio of more than 30. In order for a fiber to act as an effective reinforcement for polymers, stress transfer from the polymer matrix to the fibers is essential [25, 27]. This is achieved by having a fiber length equal to or greater than the critical fiber length and the given fiber aspect ratio in the range of 30–94 [27]. It has also been concluded that for advanced fiber reinforced resin composites, the critical fiber length could be as much as 50 times the diameter of the fiber. The diameter of glass fibers used in this study was 6 µm and the critical fiber length should be, therefore, around 300 µm. Deteriorated or initially poor adhesion between the fibers and polymer matrix increases the critical fiber length. Sufficient adhesion between fiber and matrix provides good load transfer between the two ingredients, which ensures that the load is transferred to the stronger fiber and this is how the fiber actually works as a reinforcement. However, if the adhesion is not strong and if any voids appear between the fiber and the matrix, these voids may act as initial fracture sites in the matrix and facilitate the breakdown of the material. That is why adhesion between the fibers and the resin matrix is significant for the mechanical performance and the longevity of restorations [27]. In this experimental SFRC microfibers were exceptionally well wetted with the resin (Fig. 8b) and good adhesion of the fibers most likely explained the good reinforcing effect although the aspect ratio was lower than that of earlier formulations of packable SFRC (everX Posterior, GC Corp, Tokyo, Japan).

Among the investigated conventional bulk fill resin composites, Estelite showed high values of flexural strength and modulus, which seems to be a result of high filler load level (Table 1). The most important and extensively investigated variable for physical performance in dental resin composites is filler loading. Previous studies found a positive correlation between filler loading and flexural performance [28, 29]. Kim et al. reported that the threshold of filler loading for the highest fracture toughness values in resin composites was 55% by volume [29]. This percent of filler loading is more important than weight percent. In this study, SDR resin composite had the lower filler loading that is 44% by volume showed better fracture toughness values than Tetric and Estelite resin composites which have volume filler loading of 46% and 56%, respectively (Table 1). In other words, this study demonstrated the absence of a direct relationship between volumetric content of inorganic particles and fracture resistance parameters (fracture toughness and flexural strength). The difference in fracture toughness and flexural properties values among the tested resin composites may be due to other factors than filler loading. Stress transfer from the polymer matrix to filler particles is one of the important factors affecting fracture toughness and flexural strength values. There may be differences in the adhesion between filler particles and matrix among these resin composites. Besides the filler system, monomer structures of the resin matrix also influence the mechanical properties.

The overall volumetric shrinkage during polymerization can be measured by dilatometer. This provides average shrinkage figures and gives reliable results for isotropic materials that have the same material properties in all orientations, such as conventional dental resin composites. This study showed that new flowable SFRC had a comparable volumetric shrinkage value (3.3%) to other tested materials (Fig. 4).

In some clinical situations, the light guide tip cannot be placed in close contact with the restoration surface. Therefore, any increase in the depth of cure obtained by curing should be considered important for daily clinical practice. Interestingly, the curing depth of SFRC was found 5 mm which is similar to other tested flowable bulk fill resin composites except Filtek Bulk Fill which had the lowest depth of cure (4 mm) (Fig. 5). This is in line with degree of conversion results, where Filtek resin composite showed the lowest DC% values (Fig. 3). This could be attributed mainly to the composition of the materials which influences the translucency and as a result the energy density which reach the lower layers of the materials.

Other factors that may influence depth of cure are shade of resin composite, type of curing unit and method of curing, all are widely discussed in the literature [30, 31]. Le Bell et al. have shown that fiber-reinforced composites conduct and scatter the light better than conventional resin composite [32]. Moreover, the light scattering and absorption coefficients of resin composites, which affect the light distribution, should also be taken into consideration.

Despite the higher inorganic filler content of Estelite Bulk Fill resin composite, it exhibited significantly higher amount of water sorption compared with the other resin composites tested in this study (Fig. 6). The water sorption of resin composites is mainly affected by hydrophilicity and cross-linking of the network structure. In addition, the porosity and the nature of the filler and filler matrix interface play a role in the amount of uptake during the exposure time [5].

The wear of resin composite is a complex process involving fatigue, as well as erosive, adhesive, and abrasive components [33]. The two-body wear test has been developed to simulate in vivo wear and many authors have used, though a high variation in the results have been seen even with the same material and testing technique [33]. In our study, the lowest wear depth values were found for SFRC and Tetric EvoFlow resin composites (18.2 and 19 µm) (Fig. 7). Thus, microfiber filler loading was not worsening the wear of the SFRC resin composite. This is in agreement with a study by Suzuki, who evaluated the wear resistance of commercial short fiber-reinforced resin composite (Alert) in comparison with different resin composite materials after 400,000 cycles with load of 75 N using enamel as antagonist [34]. Interestingly, the wear values of short fiber resin composite and enamel in his study were comparable to other tested resin composite materials and none of the tested materials exhibited a very coarse, worn surface after the test. Therefore, he concluded that short fiber resin composite fulfill the ADA criterion for wear. In another study, Wang and his colleagues evaluated the wear resistance and surface roughness of Alert resin composite after a simulated tooth brushing test (100,000 cycles) [35]. They showed that short fiber resin composite has similar wear resistance and surface roughness than conventional resin composites. In line with this, Dijken and Grönberg demonstrated satisfactory clinical performance (up to 6 years) of commercial short fiber resin composite (Alert) in Class II cavities [36]. They did not report any remarkable incidence of wear or loss of proximal contact with composite restorations made from Alert [36]. The surfaces inside the wear facets of the flowable SFRC were relatively smooth, similar to that of commercial bulk fill resin composites used (Fig. 8c, d). The protrusion of microfibers was not observed, and instead of the fibers being pulled out to produce a pitted surface, the fibers were microfractured into small pieces and were polished down together with resin matrix.

According to the results obtained in this work, the new flowable SFRC tested may be used efficiently as a restorative material in stress bearing areas. However, it is necessary to emphasize that this is the first study introduced data of a new short fiber reinforced flowable resin composite and to acknowledge the results obtained with the present study, this should be followed by other laboratory research and long-term clinical studies to assure the materials performance under normal clinical conditions.

## Conclusions

Within the limits of this in vitro study, it can be concluded that commercial flowable bulk fill resin composites have different properties, which should be taken into account when optimum clinical results are to be achieved. The new short fiber-reinforced flowable resin composite (SFRC) revealed improved fracture toughness compared with the flowable bulk fill resin composites. This could suggest better performance of SFRC in high stress-bearing application areas.
